# Stabilization of RNA through Absorption by Functionalized Mesoporous Silicate Nanospheres

**DOI:** 10.1371/journal.pone.0050356

**Published:** 2012-11-30

**Authors:** Brandy J. Johnson, Brian J. Melde, Michael A. Dinderman, Baochuan Lin

**Affiliations:** Center for Bio/Molecular Science and Engineering, Naval Research Laboratory, Washington D.C., United States of America; Clarkson University, United states of America

## Abstract

The potential for encapsulating RNA within tunable, semi-permeable structures for storage and transportation purposes offers an interesting approach to the reduction of stringent storage requirements that often hamper the field application of genetic analysis methods. In this study, we assessed the potential for application of functionalized, porous silicate sorbents in maintaining nucleic acid integrity. Mesoporous silica nanoparticles (MSNs) with and without incorporated stabilizing reagents were used to encapsulate triosephosphate isomerase mRNA of *Arabidopsis thaliana*. The absorption, elution, and the long-term stability of the RNA were monitored by using quantitative real-time RT-PCR. The results indicate that adsorbed RNA can be eluted from the sorbents using simple buffers and employed directly for downstream molecular diagnostic assays without any further processing. RNA integrity can be maintained for extended time periods under refrigeration temperatures in the presence of covalently immobilized stabilizing compounds. This study provides initial evidence of the potential for application of MSNs in transportation and storage. They may also have utility in sample collection and processing in restrictive environments.

## Introduction

With the advancement of molecular diagnostic technologies, detection of nucleic acid targets has been increasingly applied in medical, biological, environmental, and food-related diagnostic assays [1]. To ensure the reproducibility and accuracy of the molecular assays, there is a need to maintain the nucleic acid integrity from sample collection, transportation, and processing to storage. Traditional methods require ultra low-temperature, *i.e.* −20°C or −80°C freezers or liquid nitrogen, during these processes [2]. RNA in particular is much more labile than DNA and can be hydrolyzed readily when exposed to conditions of high pH, metal cations, or high temperatures, as well as contaminating RNA ribonucleases (RNases). RNases are known to be present endogenously in cells, tissues, body oils, and bacteria and/or fungi in airborne dust particles, the main concern for preserving the integrity of RNA [3]. There are a number of commercial products for preservation during sample collection: RNA*later* Tissue Collection: RNA Stabilization Solution (Life Technologies, Carlsbad, CA ), RNA*later* RNA Stabilization Reagent (Qiagen, Valencia, Ca), PAXgene tubes (PreAnalytix, Valencia, CA), and RNAstable® (Biomātrica, San Diego, CA). Alternatively, RNA can be protected within a physical barrier employing materials similar to those used in DNA encapsulation: liposomes, micelles, or polymers [4]–[7]. RNA encapsulation methods developed have mainly been used as delivery system for small interfering RNA (siRNA) [4], [5], [7]–[9]. In contrast, the potential of methods utilizing encapsulation of RNA within tunable, semi-permeable structures has not been fully explored for stabilization and storage purposes. Materials that allow small molecules to diffuse through while limiting the diffusion of proteins, *i.e.* RNases, can provide ideal environments to prevent RNA degradation resulting from exposure to chemicals or enzymes which in turn provide the potential for storing and transporting nucleic acids at room temperature in a cost-effective, environmentally friendly manner.

One example of these types of structures is mesoporous silica nanoparticles (MSNs). These materials offer high surface areas and ordered or semi-ordered pore structures. Similar to the MCM-41 mesoporous silicate, MSNs are often synthesized with surfactant templates to provide surface areas of up to 1,000 m^2^/g and ordered pore systems with narrow pore size distributions. Reaction conditions can be chosen to yield relatively monodisperse particle sizes (50–200 nm). Small particle sizes allow for capping of mesopores or other modifications that may prevent undesired release of encapsulated cargo. The nanoparticle morphology also offers advantages in adsorption rates and saturation loading levels [10]–[13]. These features have been applied to many studies of MSN materials related to biosensing and controlled delivery [14]–[17]. Materials of this type have been shown to provide stability to proteins through adsorption interactions as well as through covalent immobilization [18], [19].

In this study, we demonstrated successful adsorption of RNA using silicate nanoparticles. The adsorbed RNA can be eluted from the sorbents using simple buffers and employed directly for downstream molecular diagnostic assays without any further processing. We also evaluated the long term stability of the encapsulated RNA. The results indicate that RNA integrity can be maintained for extended time periods under refrigeration temperatures when adsorbed in the presence of covalently immobilized stabilizing compounds. This preliminary study assesses the potential for application of tailored, functionalized, porous silicate sorbents in maintaining nucleic acid integrity. The goal is to extend the functional lifetime of reagents and samples under less than ideal conditions. Possible applications range from sample collection, transportation, and processing to extended storage or storage in restrictive environments.

## Materials and Methods

### Chemicals

Bovine serum albumin (BSA), trehalose, glucosamine, tetraethyl orthosilicate 98% (TEOS), mesitylene 98% (TMB), and hexadecyltrimethylammonium (*i.e.* cetyltrimethylammonium) bromide (CTAB) were obtained from Sigma-Aldrich Corporation (St. Louis, MO). 3-Aminopropyltrimethoxysilane (APS) and 3-isocyanatopropyltriethoxysilane (ICS) was obtained from Gelest, Inc. (Tullytown, PA). 1-Ethyl-3-[3-dimethylaminopropyl]carbodiimide (EDC) was purchased from Pierce Chemical Company (Rockford, IL). NaOH (1N solution) and hydrochloric acid 37.6% were obtained from Fisher Scientific (Pittsburg, PA), and 200 proof ethanol was from the Warner-Graham Company (Cockeysville, MD). Chemicals were used as received. Deionized water (18.2 MΩ-cm) was obtained using Milli Q UV-Plus water purification system (Millipore, Billerica, MA).

### Synthesis and characterization of mesoporous silica nanoparticles

**Figure 1:**
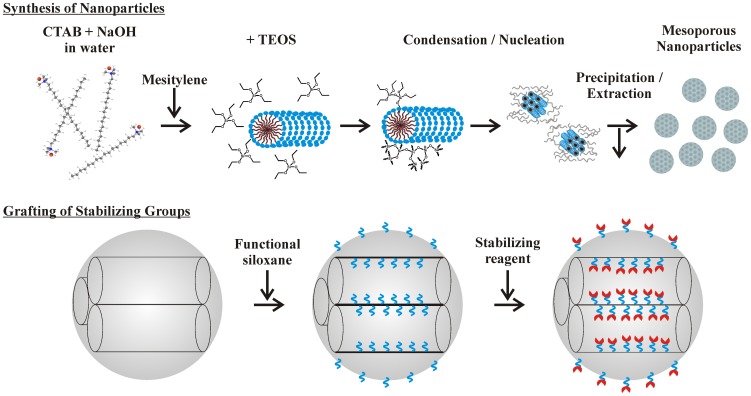
Schematic representations of synthesis and functionalization of nanospherical silicate particles.

Synthesis was adapted from a published procedure (**Fig. 1**) [12]. Briefly, 1.0 g of CTAB was dissolved at 80°C in 475 mL water and 7.0 mL 1.0 M NaOH with stirring. The reactor vessel was a polyethylene bottle suspended in a temperature controlled water bath. Mesitylene (6 mL) was added to the stirring surfactant solution. TEOS (5.0 mL) was added drop-wise, and a white precipitate formed. The mixture was stirred and heated at 80°C, collected by filtration, and allowed to dry at room temperature. As-synthesized material was refluxed in 160 mL of ethanol with 5 mL of concentrated HCl over night. MSNs were separated from the acidified ethanol by centrifugation. They were suspended in ethanol, centrifuged, and resuspended three times in H_2_O followed by centrifugation each time. Extracted MSNs were dried at 80°C.

Nitrogen adsorption experiments were performed on a Micromeritics ASAP 2010 porosimeter at 77 K (Micromeritics Instrument Corporation, Norcross, GA; Figure 1A). Samples were degassed to 1 µm Hg at 100°C prior to analysis. Surface area was determined to be 730 m^2^/g by use of the Brunauer-Emmett-Teller (BET) method; average pore size (50 Å) was calculated by the Barrett-Joyner-Halenda (BJH) method from the adsorption branch of the isotherm; and total pore volume (0.750 cm^3^/g) was determined by the single point method at relative pressure (P/P_0_) 0.97. Scanning electron microscopy (SEM) samples were mounted on SEM stubs using conducting carbon tape. Sputter coating with gold under argon was accomplished using an auto sputter coater (Cressington 108) for a duration of 60 s. SEMs were collected using a LEO 1455 SEM (Carl Zeiss SMT, Inc., Peabody, MA).

Modification of the silicate structure by stabilizing compounds was accomplished by first providing functional groups on the silicate surface (Fig. 1). Materials (1 g) were refluxed with the appropriate precursor (APS or ICS; 22 mM) in toluene overnight [20]. Functionalized materials were recovered using vacuum filtration with Whatman #5 filter paper, rinsed with toluene, and dried at 110°C. For immobilization of sugars, the ICS functionalized sorbent (1 g) was placed in solution with an excess of the sugar (1 g in 0.25 L). The solution was then mixed for 48 h before the material was recovered by vacuum filtration, thoroughly rinsed with deionized water to remove excess, unbound sugar, and dried at 60°C for 24 h. For immobilization of BSA, EDC chemistry was used. APS functionalized silicate material (1 g) was placed with 1 g BSA in a solution of 5 mM EDC in 100 mM MES buffer (2-(*N*-morpholino)ethanesulfonic acid; pH 5.5). The solution was incubated with agitation overnight, rinsed thoroughly with water, and dried at 50°C for 24 h. From this point, all solutions coming into contact with the materials were prepared using nuclease free water.

### Control RNA

Triosephosphate isomerase (TIM, GenBank accession no. AF247559) of *Arabidopsis thaliana* was chosen as control RNA. A 1061 nucleotides segment of the gene was PCR out of cDNAs of *A. thaliana* and cloned into TOPO4.0 vector (Life Technologies, Carlsbad, CA). The plasmid containing the TIM gene was used as template for PCR with M13 primers, then digested with restriction enzymes PstI and XbaI (New England BioLabs, Inc. Ipswich, MA), then cloned into pSP64 polyA Vector (Promega Corporation, Madison, WI) digested with the same enzymes to generate pSP64poly(A)-TIM. TIM RNA transcripts were generated from pSP64poly(A)-TIM linearized with EcoRI and in vitro transcribed from the SP6 promoter using the MEGAscript high-yield transciption kit (Life Technologies) according to the manufacture's recommended protocol. RNA labeling reactions were carried out in 30 µL reaction volume containing 2 µg of TIM RNA using ULS™ fluorescent Labeling kit for Agilent arrays with Cy3 (KREATECH Biotechnology, Amsterdam, the Netherlands) according to the manufacture's recommended protocol. Cy3-labeled RNA was stored at −20°C freezer until ready to be used. A NanoDrop 2000 spectrophotometer (Thermo Scientific, Wilmington, DE) was used for analysis of Cy3-labeled RNA. Microarray mode was used with analysis correction at 340 nm and automated parameters for RNA and cy3. Label analysis indicated a labeling ratio of 15 cy3 to RNA copy.

### RNA adsorption

Adsorption of RNA by the porous sorbents was evaluated by placing a fixed quantity of sorbent in aqueous solution (total volume 60 µL) with a known concentration of TIM RNA in a 0.5 mL Eppendorf tube. Samples were briefly vortexed and placed on an agitator. Initial samples were incubated for varying time periods from 10 min. to 3 h. with agitation. Increased incubation time did not result in an increase in the RNA adsorbed. All data presented here are the result of a 10 min incubation. Following incubation, samples were centrifuged at 2,500 rpm for 5 min., and supernatants were separated from the precipitated sorbents. This protocol was followed for samples used in PCR analysis as well as in fluorescence analysis.

### RNA elution

RNA elution was performed using 20 µL of EB buffer (10 mM Tris-Cl, pH 8.5) at room temperature. NS with encapsulated RNA were mixed with EB buffer and vortexed briefly to mix, then incubated at room temperature for 10 minutes. After incubation, the samples were centrifuged at 2,000 rpm for 10 min.; the supernatants were used for quantitative real-time reverse transcription-PCR (qRT-PCR). Other elution buffers, such as nuclease free water and NEB RNA elution buffer (20 mM Tris-Cl, pH 7.5 with 1 mM EDTA, New England BioLabs Inc.), were also used. For NEB RNA elution buffer, the sorbents were incubated at 50°C for 10 min.. For nuclease free water, sorbents were incubated at either room temperature or 65°C for 10 min. Alternatively, the nuclease free water was heated to 95°C then added to sorbents, and incubated at room temperature for 10 min.

### One-step quantitative real-time RT-PCR (qRT-PCR)

qRT-PCR was performed using iScript™ one-step RT-PCR kit (Bio-Rad Laboratories, Hercules, CA) with primers TIM134F (5′-CCGCCGTCTCCTCCCACCAA-3′) and TIM252R (5′-TCCGGATCCAGCCATGGCAAC-3′). One µL of eluted RNA was used as template for 25 µL RT-PCR reactions using MyiQ Thermal Cycler (Bio-Rad Laboratories). 10× serial diluted TIM RNA (1–10^5^ fg/µL) were used as standard curve. The qRT-PCR reactions were performed using the following conditions: 50°C, 10 min, and 95°C for 3 min 30 sec, followed by 30–35 cycles of 10 sec of denaturing at 95°C; and 20 sec of annealing/extension at 64°C.

## Results

**Figure 2:**
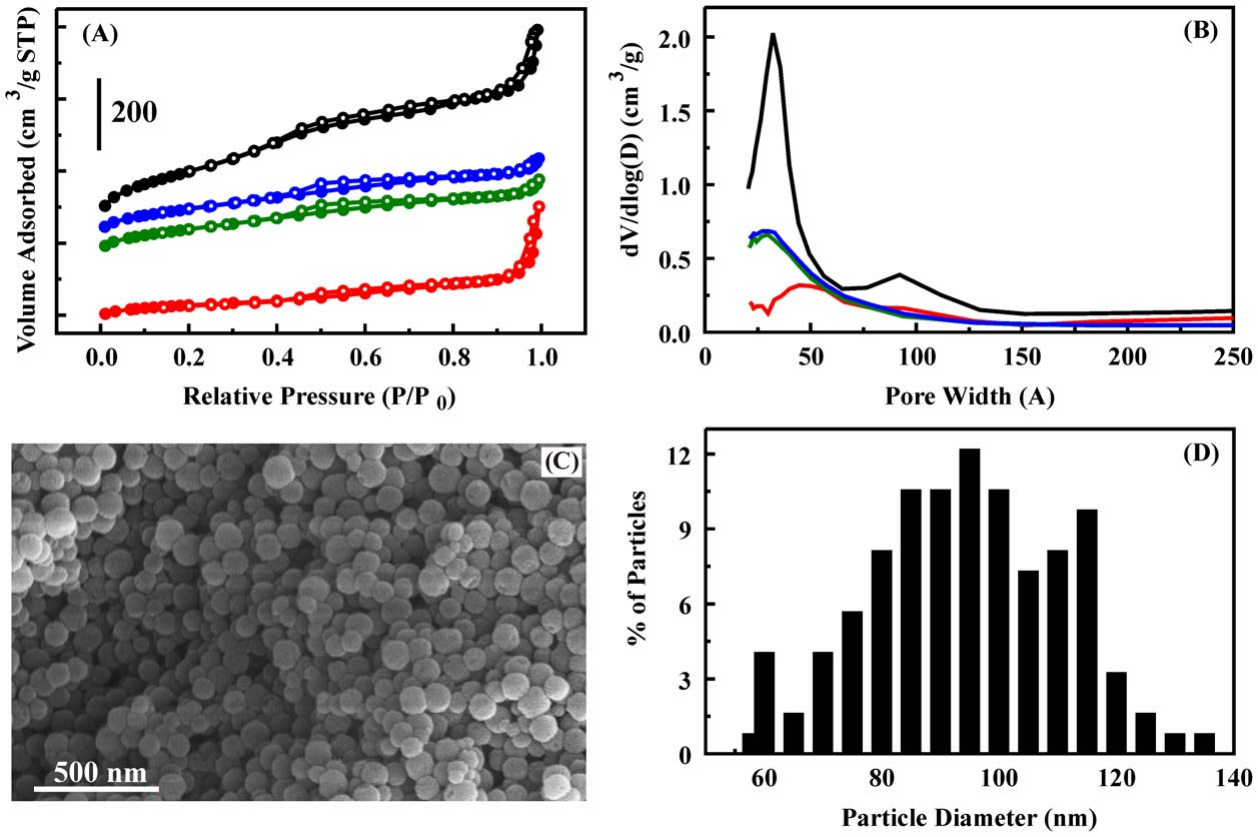
Characterization of nanospherical silicate particles by nitrogen adsorption and SEM imaging: NS (black), NS-G (blue), NS-T (green), and NS-B (red). **A.** Nitrogen adsorption/desorption isotherms. **B.** Pore size distributions. **C.** SEM image. **D.** Particle diameter distribution determined from SEM images.

The support material generated for this study has a surface area of 730 m^2^/g and a pore volume of 0.75 cm^3^/g. Nitrogen sorption characterization yielded a type IV isotherm with a steep increase in adsorption in the relative pressure range (ca. 0.2–0.45), corresponding to capillary condensation in channel-type mesopores (**Fig. 2**). Another adsorption increase was observed in the high pressure region near P/P_0_ = 1.0, which may be due to textural porosity formed by aggregated nanoparticles or other larger mesopores [12], [21]. The pore size distribution shows two peaks at 32 and 92 Å with an average pore size of 50 Å (**Fig. 2**). Larger mesopores have been noted to result from incomplete wall formation on the outermost layer of some particles where entrances appear to be shared by two or more of the smaller mesopore channels [12]. This could account for the minor and relatively wide pore size distribution peak around 92 Å. This feature may also be the result of inhomogeneous incorporation of mesitylene between CTAB micelles due to mixing conditions. Similar reaction conditions have been reported to produce a material having a bimodal pore size distribution with peaks at 54 Å and 145 Å when the amount of mesitylene was increased (7 mL instead of 6 mL). It should be noted that changes in the reactor vessel and the method of temperature control have also been observed to result in varied pore sizes and distributions. The particles are spherical with an average diameter of 96 nm (determined from SEM images, **Fig. 2C**).

Sorbent materials (NS) were functionalized with trehalose (NS-T), glucosamine (NS-G), and BSA (NS-B). Functionalization of sorbents resulted in a loss in surface area for the NS-G and NS-T materials to 320 m^2^/g with an accompanying loss in pore volume to 0.31 cm^3^/g. Reductions in surface area and pore volume were greater upon BSA functionalization (166 m^2^/g and 0.26 cm^3^/g). The pore size distributions also showed changes with a loss in definition of the peak at 92 Å for glucosamine and trehalose functionalization (**Fig. 2B**). BSA functionalization resulted in an apparent loss in smaller diameter mesopores. The differences in the materials noted here are not unexpected. BSA is significantly larger than the other compounds that were evaluated. The more significant decrease in measured surface area and pore volume for the BSA functionalized sorbent results from a greater occupation of pore volume by this compound.

### RNA absorption

**Figure 3:**
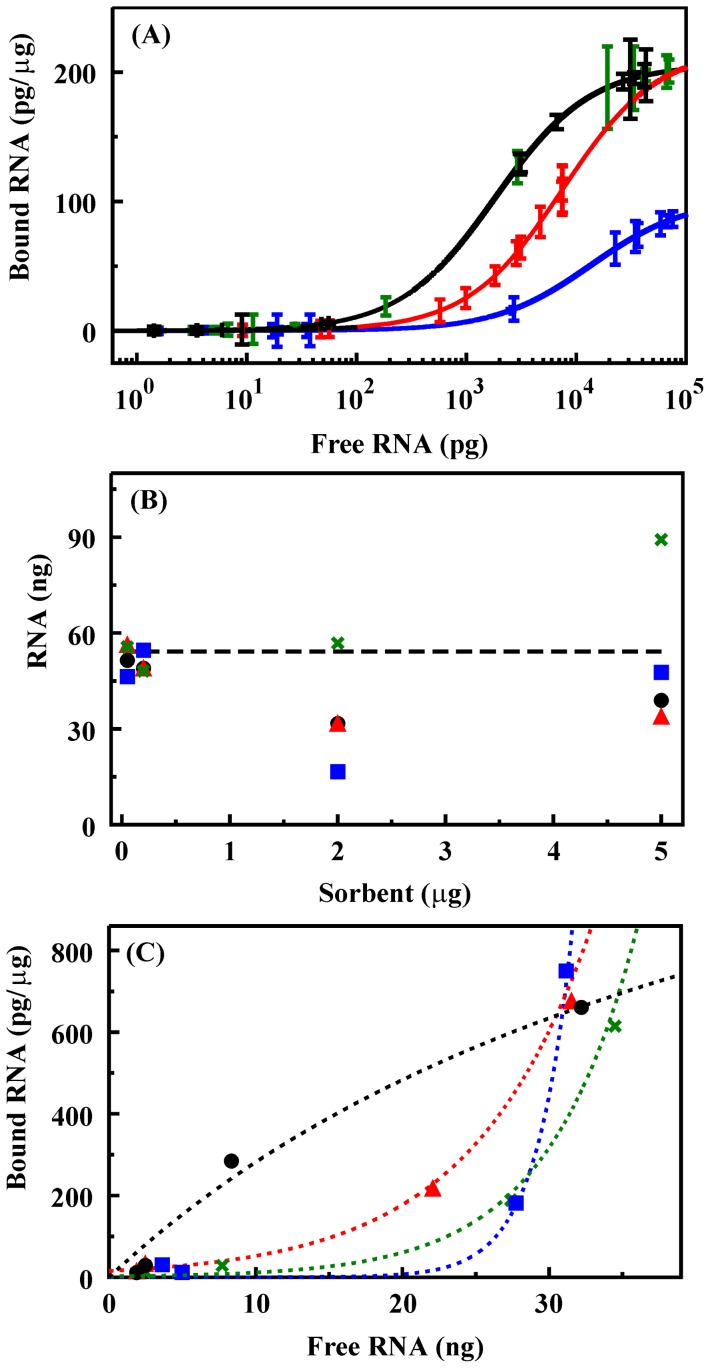
Binding of RNA by silicate materials. **A.** Binding isotherms for NS (black line, black symbols), NS-T (black line, green symbols), NS-G (blue), and NS-B (red) determined based on results of RT-PCR. Error bars are the standard deviation in the measurements. **B.** Comparison of initial RNA concentration to that recovered based on the fluorescence intensities of the supernatant and the solid components of the experiment: applied (dashed line); NS (black), NS-T (green), NS-G (blue), NS-B (red). **C.** Binding isotherm based on fluorescence intensity of the supernatant: NS (black), NS-T (green), NS-G (blue), NS-B (open circle). Lines are provided to indicate trends and are not representative of fits for this data.

RNA adsorption isotherms were determined for the four materials variants (**Fig. 3A**). Equilibrium adsorption for all sorbents was reached within the first 10 min of contact with RNA containing solutions (data not shown). The Langmuir-Freundlich (LF) binding isotherm is a generalized form of the Langmuir model often applied to solid sorbents. It allows for calculation of an association constant for the target (*k*), the saturated loading capacity of the sorbent (*q_s_*), and the site heterogeneity (*n*) within the sorbent based on the free ([*L*], pg) and bound target (*q*, pg/µg) [22]–[24].
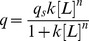



We defined criteria requiring a difference of greater than three times the standard deviation in the measurements over a range of no less than five consecutive data points as an indicator of uniqueness in the data sets. Differences in adsorption of RNA by the NS and NS-T materials were within the noise of the measurements and failed to meet these criteria. The data sets for NS-B and NS-G sorbents met the criteria for uniqueness. Fitting of the data using the LF binding isotherm indicated a saturation capacity of 205 pg/µg for the NS and NS-T materials. The saturation capacity for NS-B was similar at 219 pg/µg and slightly less for NS-G at 102 pg/µg. Affinity constants for NS and NS-T (5.5×10^−4^ pg^−1^; standard deviation 1.8×10^−5^) were greater than those determined for NS-B (1.3×10^−4^ pg^−1^; standard deviation 8.5×10^−6^) and NS-G (7.3×10^−5^ pg^−1^; standard deviation 1.9×10^−9^). Fits of the data indicated homogeneous interaction sites within the sorbents (n = 1 in all cases).

In addition to evaluating RNA binding based on PCR analysis, we attempted to evaluate binding using fluorescently labeled RNA (see Materials and Methods). Experiments were identical to those used in PCR based analysis. The supernatant and retained solid portions of the experiment were analyzed separately, and RNA content was determined based on comparison of the fluorescence intensity (550 nm excitation, 570 nm emission) of the samples to a control curve generated using varied RNA concentrations. The fluorescence intensity of the RNA bound to the sorbent did not follow the concentration curve generated in solution. This variation was identified based on the fact that the calculated concentrations for the supernatant and solid for a given sample did not total the initial concentration used in the experiments (**Fig. 3B**). The variation was different for the different sorbents. While NS, NS-G, and NS-B variations indicate less target recovered than the initial value, NS-T shows apparently more than the initial target concentration. NS-G shows a larger deviation than that observed for NS or NS-B. Additional analyses based on the results obtained for the supernatant only were also not informative. Binding isotherms observed based on PCR analysis (above) followed the form expected for binding of targets to the surface of a sorbent (LF isotherm). When binding was analyzed by fluorescence, the form of the isotherms, with the exception of the NS sorbent, strongly deviated from this formalism (**Fig. 3C**). In addition to the strong deviation from the LF isotherm, bound RNA was much higher than that predicted based on the PCR results.

### RNA elution

**Figure 4:**
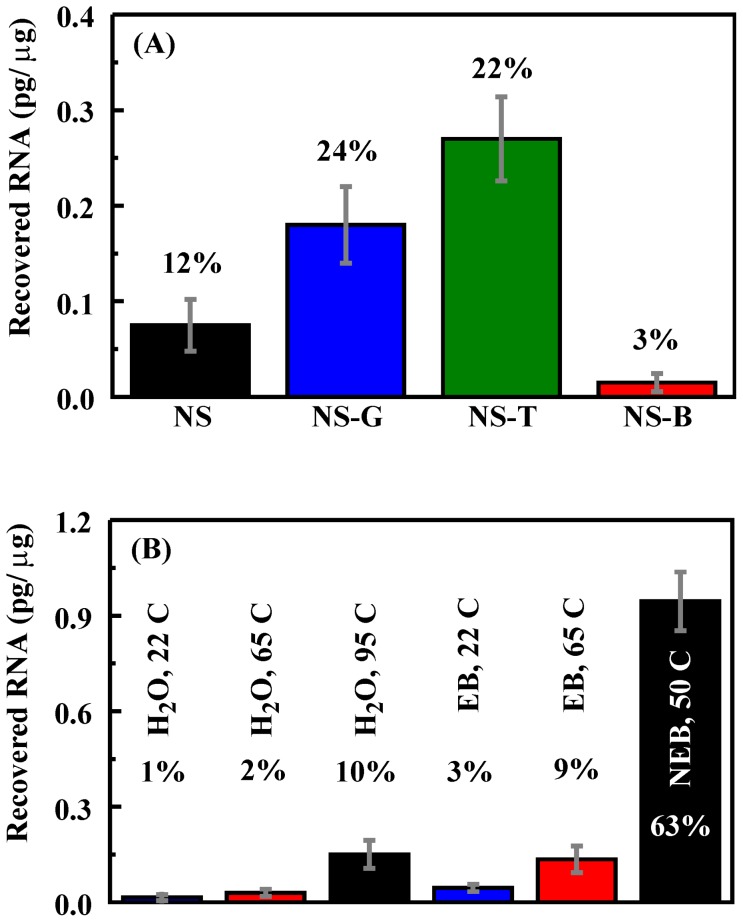
Recovery of RNA adsorbed by sorbents. **A.** Recovery of RNA from sorbents using EB buffer for elution at room temperature (22°C). Total applied target for these studies was 1.5 pg/µg. **B.** Recovery of RNA from NS-B using varied eluents at different temperatures. Values indicate the percentage of the total target bound that was recovered in the elution step.

Loading conditions were selected so that all four materials adsorbed greater than 90% of the RNA applied (1.5 pg/µg). We first evaluated the recovery of bound RNA using EB buffer (10 mM Tris-Cl, pH 8.5) at room temperature. Recovery of RNA from NS-B (∼1%) was lower than that for any of the other sorbents (5–18%; **Fig. 4A**). In an attempt to improve this recovery rate, various elution solutions and temperatures were evaluated. Because NS-B yielded the poorest RNA recovery, it was used as the model material for these studies. NEB RNA elution buffer (New England BioLabs, Inc.) and nuclease free water were selected as eluent solutions in addition to the EB elution buffer. Results indicated that recovery of RNA in nuclease free water was comparable to EB buffer (∼1%). Increasing the incubation temperature to 65°C improved the recovery rate (∼2%), and heating nuclease free water to 95°C improved the recovery rate further (∼10%). Increased incubation temperature similarly improved the recovery rate for EB buffer. The most effective recovery was achieved using NEB RNA elution buffer at 50°C (63%; **Fig. 4B**).

### RNA stability

**Figure 5:**
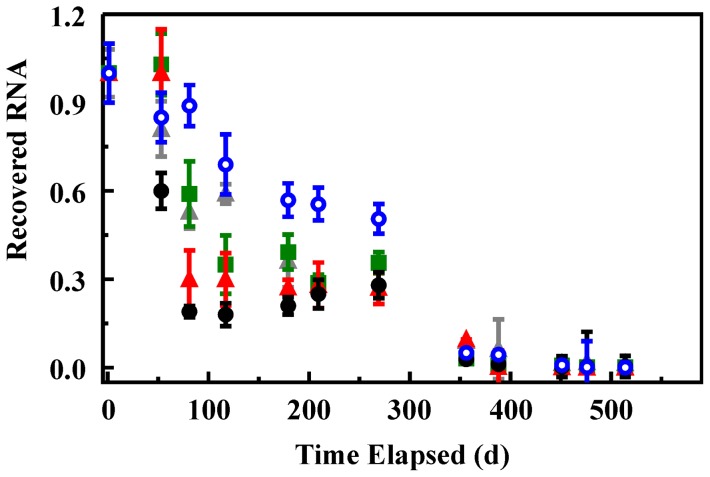
Recovery of RNA adsorbed onto sorbents following storage at 4°C. Data is presented as the ratio of the RNA recovered on a given day to that recovered for the same sorbent on day one of the experiment. Recovery of RNA from NS (black), NS-G (blue) NS-T (green), and NS-B (red) sorbents. An RNA control in water under identical storage conditions was also monitored over this time period (gray).

Adsorption of RNA to the silicate sorbents described here is intended to provide interactions that improve the stability of the RNA. Retained RNA from that initially adsorbed to the sorbents was evaluated following storage at 4°C over a period of 16 months. Because these studies were initiated prior to completion of studies on varied elution conditions, extraction of the RNA into EB buffer at room temperature was utilized. Multiple samples for each sorbent were prepared as described for the adsorption studies. The supernatant was removed from the samples, but they were not dried; adsorbed RNA on the sorbents was stored in the presence of residual water. RNA remaining in the supernatant was evaluated using the PCR protocol described above. On day one of the trial, elution of RNA from samples of each material was evaluated. The amount recovered in this experiment was used to normalize recovered amounts on subsequent days in the experiment. All recovered values are for individual sacrificial samples taken from those prepared at the initiation of the experiment (**Fig. 5**). Early results (53 days) indicated improved stability in all functionalized sorbent materials over that of the NS sorbent. After 81 days, however, the differences between the NS, NS-B, and NS-T materials become less significant. The NS-G sorbent consistently retained more RNA than the other sorbents through 260 days. At the one year point, all sorbents showed similar RNA retention at 2 orders of magnitude less than that recovered on day 1.

## Discussion

This preliminary study assessed the utility of functionalized porous silicate sorbents in maintaining RNA integrity with the idea of facilitating storage and transportation of the materials under less stringent conditions. The simple sorbents described here offer a first step in providing this capability. Silicate materials were functionalized with compounds expected to offer interactions with nucleic acids that would further stabilize the absorbed nucleic acids. Sugars, such as sucrose, dextran, and trehalose, are known to effectively protect nucleic acids during lyophilization and freeze-drying processes [25]. Trehalose, a nonreducing disaccharide, is also known to enhance the stability of fragile biomolecules in a dry state [26], [27]. Because of the potential for contamination of nucleic acid samples by stabilizing compounds resulting in interference with further analysis, the reagents selected here were chosen for both their potential in stabilization and because they offer functional groups that would provide sites for covalent immobilization to the silicate surface. Another consideration is the size of the pores in these materials. Large polyethylene glycol (PEG) molecules, for example, would be expected to occupy a large portion of the available pore volume and to hinder the diffusion of nucleic acids into and out of the pore structure. Given these considerations, trehalose, glucosamine, and BSA (denatured after incorporation) were selected as stabilizing reagents for our initial evaluations.

Immobilization of stabilizing reagents, with the exception of trehalose, resulted in a decrease in sorbent affinity for RNA. Glucosamine immobilization also resulted in a decreased saturation loading level while all materials were described as having homogeneous binding sites. The homogeneity of binding sites is not surprising since binding of RNA by the sorbents is through nonspecific interactions that will vary based on the orientation and concentration of the functional groups on the surface as well as on the orientation of the RNA oligomers and their proximity to one another. The affinity coefficient and homogeneity factors determined based on the LF isotherm capture averages across all of the possible interactions. The significantly lower affinity and saturation capacity of NS-G are likely due to unfavorable interactions between the sugar and RNA. Glucosamine may also interact with the surface of the sorbent in a way that prevents the interaction of RNA with that surface.

Fluorescence analysis of RNA binding and elution would have provided a method for evaluation of sorbents during continued development requiring less time and offering reduced error as compared to PCR analysis. Unfortunately, the attempt to evaluate adsorption using fluorescently labeled RNA was not successful. The binding isotherm observed based on the supernatant component of the assay tends to indicate the formation of multiple layers of target on the sorbent surface in contrast to the monolayer binding isotherm observed based on PCR analysis. It is likely that this change in behavior results from the interactions between the fluorescent labels on different RNA copies or from the interaction of fluorescent labels with the functionalized sorbents. When the adsorbed component of the fluorescently labeled RNA was analyzed as well, deviation was noted between the total applied and recovered RNA. It appears that adsorbed RNA does not obey the concentration curve established for RNA in solution. The likely causes of this variation are similar to those cited for the change in supernatant binding isotherm: fluorophore-fluorophore and fluorophore-sorbent interactions. The problems identified here eliminated the potential usefulness of fluorescence based evaluations utilizing this particular labeling technique. The technique employed resulted in a labeling ratio of approximately 15 Cy3 per RNA copy. It is possible that a reduced ratio of Cy3 to RNA achieved using an alternative labeling approach would facilitate fluorescence analysis while minimizing the impact on adsorption behavior.

A critical consideration in development of sorbents intended to stabilize RNA is the ability to recover the material from the sorbent. The intention is for these materials to provide stabilization of nucleic acids without complicating follow-on processing steps through the addition of compounds which may interfere with PCR analysis, use of gene arrays, or other genetic techniques. It may be possible to find a range of harsh conditions under which RNA can be eluted from the sorbents. Treatment with highly basic solutions, for example, will result in hydrolysis of the sorbent scaffold. The scaffold components in this case would be present in any following processes. In order to avoid this type of down-stream contamination, studies of these sorbents have been limited to simple considerations such as variations on temperature, pH, and salt concentrations. As a result the evaluated elution conditions provided less than perfect recovery of the RNA. The results do provide a demonstration of the potential for use of these mild techniques provided properly designed conditions and sorbents.

The recovery rate of RNA from materials functionalized with BSA was lower than that for the other sorbents though the affinity coefficients calculated indicate that the NS-T material offers a higher affinity for RNA. It is important to consider these materials as heterogeneous systems. An equivalent mass of the NS-T and NS-B sorbents (as used in the experiment) does not produce an equivalent concentration of interaction sites. The surface of the silicate, the immobilized protein (BSA), and the bound RNA form a complex system of interactions. As a result, the specific interactions and the impact of the elution buffers on these interactions can only be broadly considered. The NEB buffer has a higher salt concentration (20 mM) than the EB buffer (10 mM) and includes ethylenediaminetetraacetic acid (EDTA). Interaction of the RNA with the sorbent is through nonspecific mechanisms including electrostatic interactions. The surface of the silicate sorbent will have a negative charge under aqueous conditions, while the protein will provide sites of both negative and positive charge. The salt in the two buffers considered here (Tris-Cl) provides positive charge through primary amine groups. The lower pH of the NEB buffer (7.5 versus 8.5 for EB) provides a higher concentration of these charged groups (pKa∼8). This is one possible contributing factor to the increased effectiveness of the NEB buffer. The possible role for EDTA in the elution buffer is less clear. EDTA is negatively charged and is typically used for chelation of metals. Future studies will seek to optimize elution conditions to improve the RNA recovery rate.

Assessment of RNA stability indicated that the selected functionalization reagents offer improved RNA stability over the bare NS sorbent for at least three months under refrigeration conditions. No significant improvement over long term storage was observed with all sorbents showing similar RNA retention after 1 year. It is interesting to note, however, that the NS-G sorbent consistently retained more RNA than other sorbents over a 9 month period. These results indicate that this approach does have the potential to improve RNA stability. Other stabilizing reagents could provide greater benefits than those evaluated here. Furthermore, as a preliminary study, only one storage condition was tested. It is possible that variations in performance would be observed under different circumstances. Further study is needed to address these points.

Encapsulating RNA within porous sorbents offers the potential to design an ideal environment for stabilizing the molecules. These materials offer the potential for storing and transporting nucleic acids under less restrictive conditions in a cost-effective, environmentally friendly manner. This effort ultimately seeks to control detrimental interactions such as enzymatic and microbial degradation, the action of oxidizing and alkylating agents, base-catalyzed hydrolysis, and inter/intramolecular nucleophilic activity. Some of these mechanisms are controlled through the porous nature of the sorbents described, for example, the pores do not allow movement of bacteria through the sorbent structure. Other actions are limited due to the interaction of the RNA with a solid support which limits the mobility of the RNA. Overall, for stabilization of RNA it is desirable to disrupt 3D structures, control solvent interactions, provide reducing sites and chelating groups, and inhibit nuclease activity. The simple sorbents described here offer a first step in providing the types of materials needed. We have demonstrated the potential of the sorbents to adsorb RNA which can then be eluted at a later time. We have also shown that even these simple sorbents can provide enhanced RNA stability during storage under less than optimal conditions. Our ongoing effort will evaluate additional sorbent modifications as well as combinations of modifications. We will also continue to seek a method for extraction of higher percentages of the bound RNA without the need for harsh processing steps.
